# Reconstruction of acetabulum in revision total hip arthroplasty for pelvic discontinuity: report of a difficult case requiring four revision arthroplasty

**DOI:** 10.1186/s40064-016-2247-9

**Published:** 2016-05-11

**Authors:** Yasuo Kokubo, Hisashi Oki, Naoto Takeura, Kohei Negoro, Kenichi Takeno, Tsuyoshi Miyazaki, Daisuke Sugita, Hideaki Nakajima

**Affiliations:** Department of Orthopaedics and Rehabilitation Medicine, Faculty of Medical Sciences, University of Fukui, Matsuoka Shimoaizuki 23, Eiheiji, Fukui 910-1193 Japan

**Keywords:** Revision total hip arthroplasty, Reconstruction, Acetabulum, Bone grafting, Pelvic discontinuity, Surgical technique

## Abstract

**Background:**

Massive bone defects of the acetabulum with pelvic discontinuity are one of the major problems in revision total hip arthroplasty. Several techniques have been described for repair of acetabular defect; however, reconstruction of acetabulum with massive bone defect is still a major problem. We describe a patient who required four revision total hip arthroplasty during a 24-year period.

**Findings:**

The acetabulum with pelvic discontinuity was successfully reconstructed by stabilization of the posterior column with a plate commonly used for fracture treatment, and stabilization of the anterior column by reinforcement device commonly used for acetabular reconstruction. Fixation of both acetabular columns provided significant improvement of component stability.

**Conclusions:**

In the case of pelvic discontinuity with massive acetabular bone defect, reconstruction by stabilizing both acetabular columns using reconstruction plate and KT plate is one of the better surgical options.

## Background

Acetabular bone defect is one of the major difficulties in acetabular reconstruction in revision total hip arthroplasty (THA). Several techniques have been described for the repair of acetabular bone defect, including the use of cemented cup onto the structural bone graft (Paprosky and Magnus 1994), bilobed cup (Moskal et al. 2008), metal mesh (Jasty and Harris 1988), acetabular cage (Sembrano and Cheng 2008), Müller reinforcement ring (Stöckl et al. 1997), and reinforcement plate, such as Kerboull or KT plate (Okano et al. 2010; Kawanabe et al. 2007; Baba and Shitoto 2010) with or without allografting. In 120 revision THA cases conducted in our department, only two demonstrated acetabular bone defect with pelvic discontinuity. While the frequency of pelvic discontinuity in the revision THA is within a clinically acceptable range, reconstruction of the acetabulum with massive bone defect, including pelvic discontinuity, is a still perplexing problem in revision THA despite the currently available solutions. In this short communication, we describe acetabular reconstruction surgical technique for periprosthetic pelvic discontinuity.

## Case report

**Fig. 1:**
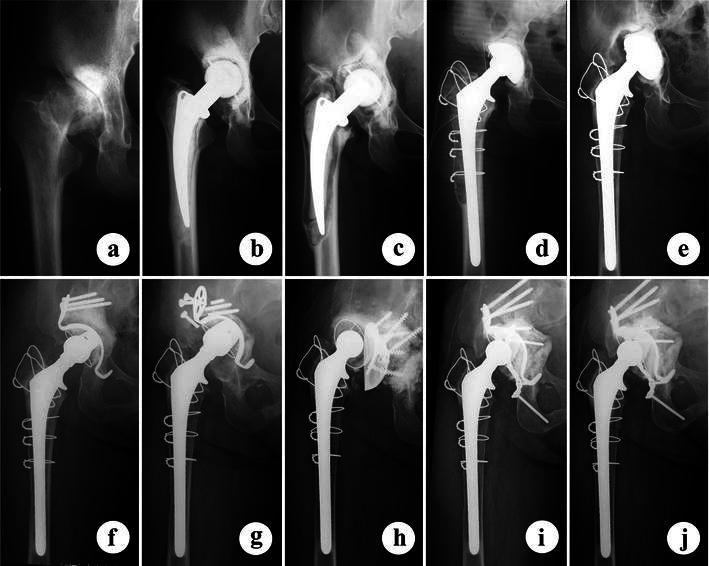
Serial anteroposterior radiographs of the patient who underwent four revision surgeries of the right hip joint. **a** Radiograph at age 39, showing dysplastic hip joint osteoarthritis; **b** after primary total hip arthroplasty (THA) at 40 years; **c** 11 years after the primary THA, showing loosening of both femoral stem and acetabular cup; **d** immediately after the first revision THA at age 51; **e** 10 years after the first revised surgery, showing acetabular cup loosening; **f** after the second revision surgery. The acetabular component was reconstructed with Kerboull reinforcement plate; **g** 3 years after the second revision surgery, showing breakage of the reinforcement plate and resorption of the graft bone; **h** after the third revision surgery using Müller ring with impaction bone grafting; **i** 3 years after the final revision at age of 72; **j** 7 years after the final revision surgery with stabilization of the two acetabular columns. Note healing of the pelvic discontinuity lesion

A 51-year-old woman was admitted to our hospital with right hip joint pain (Fig. 1a). She had undergone cemented THA 11 years earlier for dysplastic right hip joint osteoarthritis, using a Weber-Huggler-type prosthesis (Mizuho Medical, Tokyo, Japan) (Fig. 1b). Radiography showed aseptic loosening on the right THA (Fig. 1c) and the patient was advised to pursue revision THA. The femoral component was replaced with an Omnifit Specialty Hip stem (#7, 30 mm neck, 165 mm stem length, 11 mm distal diameter; Stryker Orthopaedics, Mahwah, NJ). After filling the acetabular bony defect with a saucer-like allograft, a MC1 Metal-Backed Acetabular Cup (ID: 22 mm, OD: 50 mm; Stryker Orthopaedics) was fixed with cement (Fig. 1d). Ten years after the first revision surgery, radiography of the right hip joint showed aseptic loosening of the cup with grafted bone absorption (Fig. 1e). During the second-revision THA, an acetabular bone defect of American Academy of Orthopaedic Surgeons (AAOS) classification type IIa (D’Antonio 1992) was found at the anterior to superior portion of the acetabulum. After packing a mixture of allograft bone chips and hydoxyapetite-granules, Kerboull acetabular cross plate (52 mm diameter; Stryker Orthopaedics) and polyethylene acetabular cup (ID: 28 mm, OD: 46 mm; Stryker Orthopaedics) were implanted (Fig. 1f). Three years after the surgery, right hip joint radiography showed breakage of the screws and grafted bone absorption (Fig. 1g). In the third revision surgery, a bone defect of AAOS type IV was found in the acetabulum, representing pelvic discontinuity. Reconstruction included curettage of the acetabular surface, packing a mixture of allograft bone chips and hydoxyapetite-granules, and implantation of Müller acetabular supporting ring (50 mm diameter; Zimmer, Warsaw, IN) and polyethylene acetabular cup (ID: 28 mm, OD: 48 mm; Stryker Orthopaedics) (Fig. 1h). However, 2 years after the third revision surgery, the radiograph showed dislodgement of the polyethylene acetabular cup and the Müller ring. The patient was 69-year-old and when the fourth revision surgery was performed soon after admission.

### Surgical procedure

**Fig. 2:**
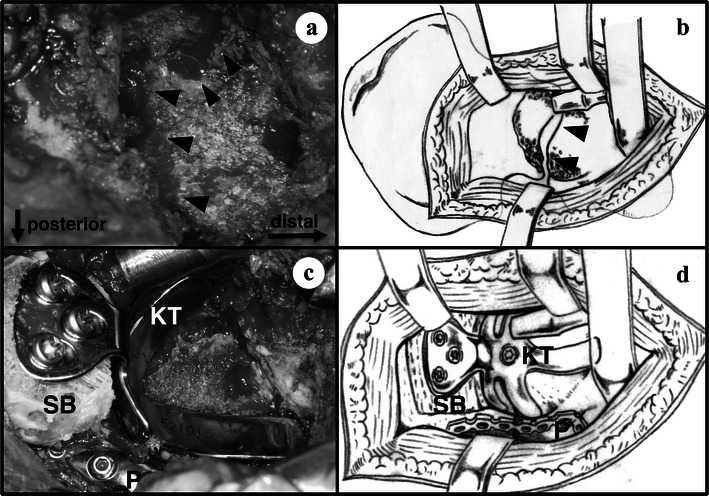
Intrapoerative photographs (**a**, **c**) and schemas (**b**, **d**) at the fourth revision hip arthroplasty. **a**, **b** Type IV bone defect of the American Academy of Orthopaedic Surgeons (*arrowheads*) with pelvic discontinuity in the acetabular surface; **c**, **d** stabilization of the acetabulum with reconstruction plate (*P*) for the posterior column and KT plate (*KT*) for the anterior column, followed by reconstruction by structural bone grafting (*SB*) at the lateral portion of the acetabulum

The posterolateral approach was adopted to view the acetabular components. After removal of the acetabular cup and Müller ring, the granulation tissue and hydroxyapatite-granules that covered the acetabular surface were curetted. A bone defect of AAOS type IV with pelvic discontinuity was identified in the acetabular surface, although bone grafting was performed in the last surgery (Fig. 2a, b). Based on this finding, the posterior column was initially fixed with reconstruction plate (Synthes, West Chester, PA), which is the same procedure applied during repair of posterior column or transverse fracture of the acetabulum (Uchida et al. 2013). Then, to stabilize the anterior column of the acetabulum and reconstruct the dysplastic acetabulum, a KT plate (52 mm diameter, 10 mm long hook; KYOCERA Medical, Osaka, Japan) was implanted with structural bone grafting between the lateral edge of the acetabulum and the plate pallet (Fig. 2c, d). Since the KT plate was very close to the acetabular surface with virtually no gap, bone grafting was not performed at the bone defect region except the lateral part of the acetabulum. Then, a polyethylene acetabular cup (ID: 26 mm, OD: 48 mm; Stryker Orthopaedics) was fixed with cement directly onto the KT plate. The patient started to walk with partial weight bearing 6 weeks after surgery, and discharged with full weight bearing walking 12 weeks after surgery. Seven years after the last surgery, the patient was able to walk with one cane but without any pain. Radiography showed stable implants and union of the pelvic discontinuity region (Fig. 1i, j). The Harris hip score was 70 at the final follow-up, which was an improvement from the score of 19 prior to the surgery.

## Discussion

Reconstruction of the acetabulum with considerable pelvic bone defects in revision hip arthroplasty requires complex surgical techniques. While bone grafting to the massive bone defects is a commonly used procedure, it is sometimes marred with failure. For examples, van Haaren et al. (2007) reported failure of the procedure in 34 % of patients with AAOS type III or IV defects that underwent revision THA during an average follow-up period of 7.2 years. They used impaction bone grafting with metal meshes to cover segmental and/or cavitary bone defect, and fixed the acetabular cup with cement on the graft bone. They argued that the reason for the high failure rate of cemented impaction grafting was related to the extent of the bone defect, particularly in case of lack of bony support behind the graft. In contrast, several other groups (Garcia-Cimbrelo et al. 2010; Ochs et al. 2008) reported a low failure rate of cemented cup with impaction bone grafting for large acetabular defects. For example, Garcia-Cimbrelo et al. (2010) described stability of the cemented cup with impaction allografting except for pelvic discontinuity. Ochs et al. (2008) concluded in their review of the literature that the incorporation rate of impacted allograft for massive bone defects depends on the use of cages or plates, and recommended complex reconstructive techniques using cages or plates for major bone defects associated with pelvic discontinuity.

The importance of cup position, which correlates with the outcome of revision surgery, was stressed in previous reports, notwithstanding operative procedure (Stöckl et al. 1997; Okano et al. 2010; Baba and Shitoto 2010; Garcia-Cimbrelo et al. 2010). A reinforcement plate or ring, such as Kerboull plate, KT plate, or Müller ring must be positioned as close as possible to the original acetabular position with morselized and/or structural bone grafting to prevent failure (Stöckl et al. 1997; Okano et al. 2010; Baba and Shitoto 2010). We experienced dislodgement of the cemented cup from the Müller ring after the third-revision THA. In this regard, Stöckl et al. (1997) described that lateral and cranial positioning of the Müller ring was associated with a high loosening rate. In addition, as we described in previous three-dimensional finite element analysis of the acetabular cup (Oki et al. 2004), the direction of the maximal resultant force acting on the hip is only about 10 degrees medial from the vertical direction in a frontal plane above the centre of the femoral head, and the shear stress on the surface of the polyethylene cup increases significantly with increases in abduction angle. We also speculated that the reason for the dislodgement of the cemented cup was the lateral positioning of the cemented cup relative to the original acetabular position, with no adequate coverage of the weight-bearing portion. This was mainly due to the large distance between the Müller ring and the host bone, the small vertical setting angle of the support ring, and instability of the region of pelvic discontinuity due to inadequate stabilization.

Pelvic discontinuity is a severe form of acetabular deficiency defined as complete separation of the superior and inferior hemipelvis. The reported rate of discontinuity encountered in revision arthroplasty ranges from 1 to 8 % of all acetabular revision s performed (van Haaren et al. 2007; Rogers et al. 2012; Gililland et al. 2013). Historically, pelvic discontinuity used to be treated by stabilization of acetabular component with bulk allografting (Paprosky and Magnus 1994). However, high failure rates of such allografting prompted various revision strategies (Gililland et al. 2013). Before the fourth revision surgery, we planned stabilizing both the anterior and posterior columns of the acetabulum similar to the treatment used for transverse acetabular fracture (Uchida et al. 2013). Gililland et al. (2013) emphasized in their biomechanical study that fixation of both columns provided significant improvement of component stability. Schwarzkopf et al. (2015) described that the use of porous metal components had very promising results because of the biological fixation. We agree with their opinion, however, it is difficult to use the cementless cup in the cases of pelvic discontinuity with massive bone defect. In our case, with massive acetabular bone defect, we stabilized posterior column by reconstruction plate, and then, stabilized anterior column by KT plate. We considered that this procedure was valuable to stabilize both columns through the single posterior approach.

## Conclusions

We described here an educational case of four-revision THA. In the case of pelvic discontinuity with massive acetabular bone defect, reconstruction by stabilizing both acetabular columns using reconstruction plate and KT plate is one of the better surgical options.
